# Oral Microbial Dysbiosis Associated with Alzheimer’s Dementia in Puerto Ricans: A Preliminary Report

**DOI:** 10.1002/alz.095310

**Published:** 2025-01-09

**Authors:** Hiram Morales, Carlos Alberto Herrero‐Rivera, Cecilia Michelle Soler‐Llompart, Ana Cecilia Sala‐Morales, Gerianne Olivieri‐Henry, Filipa Godoy‐Vitorino, Vanessa Sepulveda

**Affiliations:** ^1^ University of Puerto Rico, Medical Sciences Campus, School of Medicine, San Juan, PR USA; ^2^ University of Puerto Rico, Medical Sciences Campus, San Juan, PR USA

## Abstract

**Background:**

New studies have linked epidemiological and pathophysiological relationships between oral microbiota and Alzheimer’s disease (AD), a neurodegenerative disease more prevalent in the Puerto Rican population. Dysbiosis of the oral microbiome induces periodontal disease, which increases systemic chronic inflammation, an important component in the multifactorial pathogenesis of AD. This project aims to characterize the oral microbiota’s composition and diversity in AD patients compared to healthy controls, and explore the potential role of oral dysbiosis in dementia.

**Method:**

A total of 52 participants were recruited (28 with Alzheimer’s Disease and 24 healthy controls), IRB #2290033626. Evaluations included a complete medical history and physical exam, and psychologic assessments with the Montreal Cognitive Assessment (MOCA) and Clinical Dementia Rating (CDR). Blood samples were collected for genotyping of the APOE gene. Oral samples were collected for genomic DNA extractions and microbiome characterization using 16S rRNA genes (V4 region) via Illumina MiSeq.

**Result:**

A statistically significant difference in bacterial composition was found in beta‐diversity between AD group and Controls (p‐value = 0.027). Among cognitive stages by CDR (p‐value = 0.04), a significant difference in beta‐diversity is also seen between participants with “no dementia”, “mild dementia”, and severe dementia”, which also correlates with distinctive richness at the genus‐level. LEFSe and Correlation analysis showed an increased abundance of oral genus *Haemophilus* in controls as compared to AD, and in increase in *Corynebacterium* in AD patients. Additionally, there is an increase in the proinflammatory bacteria *Prevotella* and a decrease in anti‐inflammatory bacteria *Proteobateria* also occurs in AD participants with abundance correlating with grades of cognitive impairment.

**Conclusion:**

This research underscores the intricate interplay between oral microbiota dysbiosis, periodontal disease, chronic inflammation, and Alzheimer’s Disease (AD). Our preliminary findings suggest a potential association between alterations in oral microbiota composition and the presence and severity of AD. Further investigation, including periodontal assessments and analysis of chronic inflammatory markers, is warranted to elucidate the mechanistic links and potential therapeutic avenues in managing AD within the Puerto Rican population.

